# Empowering self-help groups for caregivers of children with disabilities in Kilifi, Kenya: Impacts and their underlying mechanisms

**DOI:** 10.1371/journal.pone.0229851

**Published:** 2020-03-09

**Authors:** Karen Bunning, Joseph K. Gona, Charles R. Newton, Frances Andrews, Chantelle Blazey, Hannah Ruddock, Jessica Henery, Sally Hartley

**Affiliations:** 1 School of Health Sciences, University of East Anglia, Norwich, United Kingdom; 2 Centre for Geographic Medicine Research (Coast), Kenya Medical Research Institute, Kilifi, Kenya; 3 Department of Psychiatry, Oxford University, Oxford, United Kingdom; 4 Sydney University, Sydney, Australia; Institute of Mental Health, SINGAPORE

## Abstract

Bringing up a child with disabilities in a low-income setting is challenged by inadequate resources, limited psycho-social support and poverty. Not surprisingly, many caregivers experience fatigue, distress and isolation. To address and investigate these issues, action was taken to set up twenty self-help groups focusing on caregiver empowerment. A realist evaluation design was adopted to evaluate impacts associated with the self-help process and to identify mechanisms determining the outcomes. Monthly monitoring visits were conducted to the groups during a ten-month set-up period, at the end of which eleven active groups remained, nine having dissolved due to disputes, corruption and extreme environmental conditions. A facilitated intervention was delivered to the active groups (N = 154) over a six-month period. The members were guided to review and discuss topics such as economic empowerment, personal situation, peer support, community inclusion, access to health and education. Evaluation employed mixed methods using questionnaires (n = 75) and semi-structured interviews (n = 36) pre- and post-intervention. At baseline, the burden of caregiving was characterised by aloneness, challenges, stigma and discrimination. Post-intervention, caregiver agency was defined by togetherness, capacity-building, acceptance and well-being. Significant impacts associated with caregiver perceptions included increased social support, reduced severity of child’s disability and decreased effects of extrinsic factors affecting the caregiver’s role. Mechanisms of ‘handling goods and money’ and ‘social ties and support’ appeared to underpin the outcomes. Caregiver empowerment was associated with newly developed skills, social connectedness and resource mobilisation. Documentation of group processes contributes to the evidence on community-based inclusive development.

## Introduction

The link between disability and poverty has been established unequivocally, with a positive relationship more likely in low-middle income countries (LMICs) of the Middle East & North Africa, and East Asia and the Pacific [1,2]. Of the 150 million children with disability worldwide, eighty percent reside in resource-poor regions of the world [3]. More recently, it was estimated that approximately 95% of 52.9 million children below 5 years with developmental disabilities resided in LMICs. This showed a lack of significant improvement to the burden of developmental disabilities compared to similar estimates in 1990 [4]. Typically, it is the mother or grandmother who performs the role of primary caregiver, often in circumstances where the husband is not present at home [5], meeting the child’s daily living needs in circumstances of limited financial resources [2], scarce information about causation [4] and poor access to rehabilitation and health care [6–9]. In many cases, the caregiver is solely responsible for the child [5,6, 10], which impacts on time for domestic duties and livelihood tasks [6, 11]. A lack of formal education and low literacy levels in African countries [11,12] has been reported, particularly amongst females in rural communities. Coping ability amongst persons living with disability was associated with better levels of education [13]. Caregiver capabilities, including educational experience and autonomous decision-making, were positively correlated with active participation in groups designed to promote self-help in Nepal [14].

Social connections and social support are positively correlated with mental and physical health, and longevity [15]. It follows, therefore, that the impoverished social networks experienced by many caregivers leave them vulnerable and alone [6, 16]. A lack of critical support from a spouse and other family members further compounds caregiver difficulties [5, 17]. Furthermore, in the community across sub-Saharan Africa, disability tends to be associated with negative images and superstitious circumstances, underpinned by various explanations [17], for example breach of social conventions in Botswana [18], Ghana [19] and Kenya [6]; and external, preternatural forces in Kenya [6, 20], Malawi [21] and Namibia [22]. Such explanations of disability give rise to stigma and discrimination, both in the immediate family and in the local community [6, 17, 19–20, 22–24]. Caregivers may be deterred from seeking help from fear of exposure [5] and may act deliberately to conceal their child from society [25]. Psychological distress, social isolation and scarce resources, combine to affect low mood, fatigue and mental health crises in some cases [26].

Self-help groups (SHGs) provide one response to the challenges experienced by caregivers. Building on the traditions of collective savings and shared livelihood activities [27,28], SHGs are identified in the ‘empowerment’ domain of the community-based rehabilitation (CBR) matrix and guidelines [29]. CBR, or its more current title, community-based inclusive development (CBID), offers a composite strategy for meeting the needs of persons with disabilities. It has been endorsed by the World Health Organisation, International Labour Organisation, UNICEF, UNESCO, and the International Disability & Development Consortium [29], The guidelines strongly emphasise ‘empowerment’ through the inclusion and participation of individuals with disabilities, their family members, and communities, in all development and decision-making processes. Initiatives in this area continue to evolve and grow in more than 90 countries worldwide, focusing on strategies for ‘rehabilitation, equalization of opportunities, poverty reduction, and social inclusion of people with disabilities’ (p.308) [3].

The specific aim of SHGs is to support people to gain control over their lives when they have been alienated from their communities or have no authority [30]. Individuals are encouraged to work together, combining their efforts to gain access to resources and critical understanding of the sociopolitical environment [31]. Founded on peer collaboration, rather than professional support [29], psychological empowerment is central to the SHG development. In an early study of leadership in grass root organisations, Kieffer [32] concluded that empowerment is about the development of skills for effective participation in community decision-making, and comprises elements of self-esteem, a sense of agency, and perceived efficacy. A few years later, Zimmerman & Rappaport reported an association between participation and perceived control that distinguished high-participation groups from low- or no-participation groups [33]. Empowerment theory has been defined according to three constructs: intrapersonal, defined as an individual’s awareness of their capacity to initiate change, the drive to have control over their circumstances and their own perceived competence; interactional, which incorporate an individual’s knowledge of the socio-political environment, and their use of skills and resources to engage with it; and behavioural, or how individuals act to bring about change in the environment [34]. Various practical interventions have adopted empowerment theory for different purposes, including: promoting the participation of persons with disability in community-based physical activity [35]; facilitating the active agency of parents in the delivery of an early intervention scheme for children with hearing impairments in India [36]; and delivering culturally appropriate, community-led empowerment training to an indigenous chid safety workforce [37]. Thus the association between empowerment and active participation is of critical relevance to the self-help process afforded by SHGs.

Self-help initiatives have been reported with a range of stakeholder groups in low and middle income countries, including caregivers of children with disabilities in Ghana [5]; mental health service users in Ghana [38] and Uganda [39]; economic and livelihood groups for women in South Asia [14, 40,41]; social support for adolescents with visual impairments in Jordan [42]. Reported benefits include financial support [38, 41]; social support and acceptance by other members of the family [5, 41,42]; growth in confidence for self-expression and reduction in domestic abuse [43] and improved family relations [44]. However, despite such positive outcomes, SHGs have been criticised for a lack of research rigour with insufficient detail on processes and activities underpinning the outcomes [40]. This resonates findings on other CBR/CBID initiatives [45,46].

A three-year project was set up to establish an effective and sustainable approach to addressing common problems experienced by caregivers of children with disabilities within their own communities. The ‘empowerment’ domain of the CBR matrix [29] was selected for its cross-cutting potential in relation to the four other domains of ‘health’, ‘education’, ‘livelihood’ and ‘social’. With the caregiver identified as the agent for change in the lives of children with disabilities, self-help groups were nominated the primary vehicle for change. In addition, there was no expectation of professional input from the already constrained rehabilitation services. The main project aim was to generate knowledge on the development process of community-based, self-help groups for caregivers and their children with disabilities in rural Kenya, that may be applicable to caregivers in similar situations in other low-income settings. Two inter-linked studies were carried out. A process evaluation investigated the implementation of SHGs (caregiver recruitment, community group support, and monitoring visits) and group factors (caregiver characteristics and start-up activities) over the 10-month set-up period, post-recruitment of caregivers. This is reported separately [47]. The current study focused on the impacts associated with caregiver participation in SHGs and the underlying mechanisms of the SHG process, through address of two research questions: 1. What changes are associated with empowering self-help groups for caregivers of children with disabilities? 2. If there are changes associated with the self-help group process, how does it do so?

## Materials and methods

The project was conducted between 2015–2018. A realist evaluation design was adopted [48] for its recognition of the different ways interventions work for different people. It was expected that the development of SHGs would be influenced by the experiences, beliefs and attitudes of the participants, the available skill mix amongst group members, and access to resources relevant to the context and environmental conditions. Mixed methods were employed for pre- and post-intervention evaluation. Structured questionnaires were administered for quantifying caregiver perceptions of their child with disabilities, their role as a caregiver and their support networks. Semi-structured interviews were carried out to contextualise the outcome measures and to provide a deeper understanding of the underlying mechanisms of the SHGs.

The setting was Kilifi County (area: 12,610Km^2^; poverty level: 71.4% [49]). It was chosen for its potential to build on existing relations with established community-based groups that had been involved in a previous study on disability awareness training [50]. Situated on the Indian Ocean coast, the inhabitants were mainly from the Mijikenda groups (about 80%) and spoke Swahili, Giriama and Chonyi. There were mixed religious practices across the area (Christianity: 70%; Islam: 10%; traditional: 20%). One of the poorest areas in Kenya, the majority of Kilifi residents lived in dwellings of mud construction consisting of one or two rooms, with no power supply or running water. Largely dependent on subsistence farming for income, per capita, the average income for a family (parents and six children) was KES1000 per month–less than USD13 [51]. Based on a county-wide population of 1,109,735 inhabitants, 50% were estimated to be children (n = 554,868). Using a 5% prevalence of childhood disability [3], it was estimated there were 27,743 children with a disability. In this setting, disability is often associated with negative images and explained by breach of social conventions by one or other of the parents, which has aroused the wrath of ancestors, supernatural forces, the will of God or unexplained events [4]. Thus stigma associated with disability was present in the community.

### Ethics

Ethical approval for the study was given by Scientific Ethics and Review Unit (SERU) in Nairobi, Kenya (SERU 0016/3132), and the International Development Ethics Committee at the University of East Anglia, UK. Participant identities were anonymised. Data were stored on a secure server with access by team members only.

### Recruitment and development

There were two distinct phases to the development of SHGs. The set-up phase focused on the recruitment of caregivers and forming of SHGs. The intervention phase focused on supporting the activities of the SHGs, monitoring progress and delivering a facilitated intervention.

- Set-up phase

The initial aim was to set up twenty SHGs across Kilifi county. Contact was made with the designated sub-chief’s office in each sub-location for early community engagement. Caregiver recruitment was carried out by women groups (WG) and community health worker groups (CHW) operating locally. All the groups had participated in a previous study on disability awareness training [50]. Each group (CHW and WG) was asked to identify around 15 caregivers of children with disabilities from their local community, making a target recruitment number of 300 caregivers. Members of the WG and CHW groups accompanied the caregivers to a first meeting for information sharing. Informed consent was solicited from those caregivers who wanted to participate in the SHG development and recorded by signature or thumbprint. Caregivers were included in the SHGs if they were: at least 18 years old; cared for a child (0–15 years) that they identified as having a primary condition affecting body function and structure, including intellectual disability, deafness, visual impairment, autistic spectrum condition, cerebral palsy, variously associated with limitations in vision, hearing, mobility, attention, learning and the effects of seizures. Exclusion criteria covered temporary disabling conditions, e.g. fractured limb, which were likely to resolve with appropriate treatment; and deficits that could be addressed through corrective devices, e.g. glasses for myopia. At the start of the set-up phase, 254 caregivers were registered across 18 SHGs–two groups having withdrawn prior to registration. During set-up, the SHGs embarked on livelihood projects for income generation, including merry-go-round (where group members contribute small sums of money or food items at each meeting, which are allocated to two or three individuals for family use and income development by rotation so each member has a turn as a beneficiary); farming; livestock rearing. The groups met every week with monitoring visits from the project researcher occurring at monthly intervals for fielding questions from the membership, supporting the groups to problem solve and providing advice as appropriate. At the end of the set-up phase, eleven groups remained comprising 154 caregivers–nine groups having dissolved variously due to drought conditions, in-group tensions, and fraudulent activity in the local community. For further information on the development process the reader is referred to Gona et al. [47].

- Intervention phase

An intervention was designed to support the focal ‘empowerment’ domain of the CBR matrix [29]. A facilitated intervention comprising six key topics: economic empowerment, sharing personal situations, peer support, community inclusion, access to health and education, was carried out. Each group received six sessions in total, one session per month delivered over a six-month period, plus a final plenary session where the group were invited to comment on the intervention. The topics were selected to support the ‘empowerment’ domain of the CBR matrix and for the correspondence to the other four domains of: livelihood, education, health and social [29]. Each was planned with its own aim, rationale and session guide, and delivered by the second author, who had conducted the monitoring visits for 12 months prior, was conversant in the local languages and familiar with the local culture. Typically, a group discussion lasted 60 minutes, with 15 minutes on reflection of the previously addressed topic and 45 minutes on the current topic. Facilitation employed pre-planned, open-ended questions, direct invitations to members to tell the group about a particular aspect of the topic, and encouragement to talk about the challenges and successes experienced. Group discussion tactics included talking in small groups and pairs before reporting back to the main group, direct solicitation of individual opinions, and inviting comments from the entire group. Any facilitation used that was additional to the planned questions was recorded in situ. During the intervention period, the groups continued to meet according to their usual frequency (i.e. once a week), but one meeting a month was assigned to the relevant topic. The required business of the group, e.g. income generating activities, continued as normal.

### Sample

A purposive-convenience sample participated in the research and was composed of 81 caregiver-participants. As shown in Table 1., the majority of the caregivers identified as ‘married’ although marital partner presence at home was not confirmed. Educational level of attainment was low with around 49% having received no formal education and 25% an incomplete primary education. Regarding numbers of children at home, 68% of the caregivers had at least 6 children, including 1 child with a disability. Just under half (46%) lived in dwellings of a poor quality (n = 37) and had either no or one type of livestock at the homestead, with 60% serving 2 or less meals per day.

**Table 1:** Summary of caregiver characteristics including quality of life indicators.

Characteristics		N	%
*Age range (years)*	<20	1	1
	21–29	15	19
	30–39	27	3
	40+	38	47
*Marital Status*	Single	4	5
	Married	56	69
	Divorced	6	7
	Widowed	15	18
*Education*	Primary–complete	18	22
	Primary–incomplete	20	25
	No formal	40	49
	Secondary	3	4
*Children at home*	1–2	9	11
	3–6	46	57
	7–10	20	25
	11+	6	7
*Children with Disabilities*	1	73	90
	2	6	7
	3	2	2
	4+	0	0
*Dwelling*	Mud & thatch–good condition	13	16
	Mud & thatch–poor condition	37	46
	Iron roof	21	26
	Permanent	10	12
*Meals served per day*	1	11	14
	2	38	47
	3	30	37
	4	2	2
*Livestock*	Chicken(s)	62	77
	Duck(s)	21	26
	Goat(s)	37	46
	Cow(s)	17	21

Of the 81 caregivers, 5 had 2 children with disabilities, with the remainder having 1 each. As shown in Table 2, there were more males (59%) than females (41%) with the majority of the children falling into the age ranges of 7–10 (30%) and 11–15 (43%). The most frequent area of difficulty identified by the caregivers was ‘physical’. Furthermore, where a single area of difficulty was identified for the children (N = 64), over half was accounted for by a physical problem (54%). Whilst physical difficulty included musculoskeletal problems such as club foot (2 cases), cerebral palsy was the major source of difficulty, which likely masked other problems in the caregiver’s report of their child’s perceived difficulties. Therefore, the figures shown for other areas may not be representative. Few disability aids were identified to be present or in use by the caregivers despite the frequency of ‘physical’ problems identified by the caregivers. Under the category ‘other’, a special seat for the child was identified in each case, although no specific detail was recorded. Less than half of the children were registered to a school or unit, with no information on actual attendance recorded.

**Table 2:** Summary of characteristics for children with disabilities reported by caregivers.

Characteristics		N	%
*Gender*	Male	51	59
	Female	35	41
*Age range*	0–3	9	11
	4–6	14	16
	7–10	26	30
	11–15	37	43
Area of difficulty identified (body function & structure)*	Vision	4	5
	Hearing	12	15
	Physical	45	54
	Drooling	2	2
	Attention	14	17
	Communicating	22	27
	Seizures	10	12
*No*. of areas of difficulty identified*	1	64	77
	2	15	18
	3	1	1
	4+	3	4
*Disability aids*	Wheelchair	0	0
	Standing frame	0	0
	Other	3	4
School registered*	Yes	35	42
	No	48	58

*missing data on 3 children

### Data collection and analysis

Two questionnaires and one semi-structured interview were administered at baseline and after the 6-month intervention period. Out of the 154 participants across the groups, the entire sample comprised 81 participants (female = 74; male = 7), who were variably involved in the three measures (two questionnaires and a semi-structured interview). Sample details are given in relation to the identified measure. Of the entire sample, only 22 participants were involved in all three measures.

Data collection was conducted at the regular SHG meeting (e.g. health dispensary, community facility, under a cashew nut tree close to the sub-location Chief’s office) by prior arrangement. The researcher and the participant sat apart from the rest of the group during administration.

- Questionnaires

The questionnaires were carried out with a comprehensive-convenience sample composed of 75 participants each, i.e. members who were present at the time of the researcher’s visit to the SHG: 1. Sub-sections of the Communication Disability Profile [52]; and 2. the Multi-dimensional Scale of Perceived Social Support [53]. Whilst different samples completed the two questionnaires, each questionnaire was administered to the same 75 participants pre- and post-intervention. It was expected that participation in the SHGs would influence the way caregivers thought about their children, their roles and their lives.

1. Communication Disability Profile (CDP) [52]: Caregiver perceptions of the severity of their child’s disability and extrinsic factors affecting their own capacity for caregiving, were captured. Adapted and shortened for an earlier study (CDP–brief version: see Bunning et al, 2014), it had been translated into Swahili and Giriama (local language) previously. Selected sections focusing on general domains rather than communication-specific content, were used. The entire first section (Body Structure/Function) was administered whereby the caregiver was invited to rate their child’s level of difficulty in 10 areas: seeing; hearing; moving; eating and drinking; drooling; paying attention; sitting still; learning; understanding; and seizures. Part of the third section (Participation) which focused on ‘Extrinsic Factors’ affecting the child and caregiver was also administered: time to perform caregiving role; support and information available; people for child to interact with; and acceptance in the local community.
2. Multi-dimensional Scale of Perceived Social Support (MSPSS) [53]: The extent and nature of social support experienced by caregivers, was assessed. The MSPSS contained 12 items, with 4 items assigned to each of 3 defined dimensions: significant other; family; friends. Not having been used in this region before, the MSPSS was translated and back-translated into Swahili and Giriama (a local language) and piloted with a sample of 18 participants prior to the main data collection.

Both questionnaires were researcher administered to accommodate limited literacy skills. Caregivers gave their response choices on a Lickert-type 7-point rating scale presented in a visual ladder-format displaying semantic descriptors in the preferred language. The ladder-scales were explained initially. After each question was read out, the participant was invited to indicate their response choice on the ladder, which was then recorded. The data were entered into a prepared Excel worksheet before importing to SPSS. The data were explored using descriptive statistics before applying the non-parametric Wilcoxon signed-rank test to each measure.

- Interviews

In order to contextualise caregiver outcomes associated with the SHG development, 36 semi-structured interviews were conducted: 18 pre-intervention; 18 post-intervention. The purposive sample comprised caregivers across all 11 SHGs, representing group members who were considered to be highly active and vocal, reserved and passive, or somewhere in the middle. The interview sample comprised 34 caregivers, of which only 2 participated in both pre- and post-intervention interviews due to the non-availability of some participants. Thus the interview data were complementary and allowed deeper understanding of issues arising at time points one and two. The second author (JKG) conducted the interviews in the home language of each participant. The questions at baseline invited the participants to reflect on: their child’s difficulties and the challenges they encountered as caregivers; the help and support received both in the immediate locality and in the wider community; recent positive and the negative experiences of their child. Post-intervention, the same questions were asked but in the context of their SHG experience (see S1 Appendix for topic guide and questioning route). Probes were used to allow caregivers to elaborate their responses more fully. The interviews were audio recorded, and later uploaded to a computer for orthographic transcription. Translation into English took place at a later stage. Queries regarding translations were managed via a process of query and back translation as appropriate.

Data analysis followed a phenomenological approach [54]. To evaluate change processes pre- to post-intervention, the framework method [55] was applied in two stages: 1. Initial thematic analysis, generation of nodes and hierarchical organisation of framework; 2. Secondary analysis involving critical review and adjustment of nodes and framework, and explication of interconnections at the levels of organising and basic themes. The data management software Nvivo-11 was used.

Stage 1 involved the first author (KB) working with two pairs of researchers in the UK. Following a process of familiarisation, the baseline data were addressed initially with each pair working through the analysis independently. Generation of a first level of nodes representing identified themes then followed, to which selected interview excerpts were assigned. The nodes were adjusted and established in a hierarchy of organising and basic themes. Once the separate analyses of pre-intervention data were completed, a comparative review was carried out until consensus was achieved. At this point a draft hierarchical framework was created for use in the. post-intervention analysis, which followed a similar process of review with changes made to the framework as appropriate. Whilst the entire data set was analysed, saturation checks were carried out at the end of stage 1. This was done by reviewing the number of references shown for each basic and organising theme in the Nvivo programme and checking that no new information had emerged, therefore obviating the need for further data collection. Stage 2 involved the first author (KB), a regular visitor to Kenya, and the second author, a citizen of the region, critically reviewing thematic assignment and labelling, textual interpretations and interconnections. In this way, cultural familiarity and a remote questioning stance were combined in the final analysis. Once consensus on the hierarchy of themes was achieved, they were organised under a pair of over-arching constructs representing opposite ends of a continuum. Data tables were constructed to capture the correspondence of different pairs of organising themes and the explicit links between organising and basic themes. Each table was checked and revised to ensure the correspondence to the identified interconnections.

### Triangulation and identification of mechanisms

Triangulation and the identification of mechanisms were addressed sequentially in a process of realist retroduction, which combined processes of deduction and induction [56]. Initially, the first and second authors critically reviewed the findings from the various data analyses for corroborating evidence of the outcomes. Next, the basic themes (under organising themes) were reviewed to determine those ones that captured the structure, activities and actions of the SHGs. These were then grouped according to their relatedness in labelled domains representing the underlying mechanisms.

## Results

### Caregiver perceptions

As shown in Table 3., the results from the two questionnaires revealed significant changes from baseline to post-intervention with large effect sizes. Caregivers rated the severity of their child’s disability as significantly less severe, indicated by a higher median score post-intervention (baseline = 68; post-intervention = 72). Extrinsic factors affecting the child and caregiver were perceived as significantly less of a problem post-intervention, similarly indicated by a higher median score post-intervention (baseline = 19; post-intervention = 29). Finally, there was significant growth in caregiver report of their social support networks (baseline = 39; post-intervention = 84). Inter-quartile ranges were generally lower for all measures at the post-intervention point, indicating reduced variability in the middle 50% of the scores.

**Table 3:** Pre- to post-intervention results for questionnaires using Wilcoxon signed rank test.

Measure	*N*	*Mdn* (pre-post)	*Inter-quartile ranges* (pre-post)	*P value*	*Effect size* (*r*)
*Communication Disability Profile*	75				
Section 1. Perceived severity of child’s disability		68–72	24–17	< .00	-.87
Section 3. Perceptions of extrinsic factors affecting caregiver		19–29	7–3	< .00	-.867
*Multi-dimensional Scale of Perceived Social Support*	75	39–84	23–6	< .00	-.84

### Caregiver experiences

**Fig 1:**
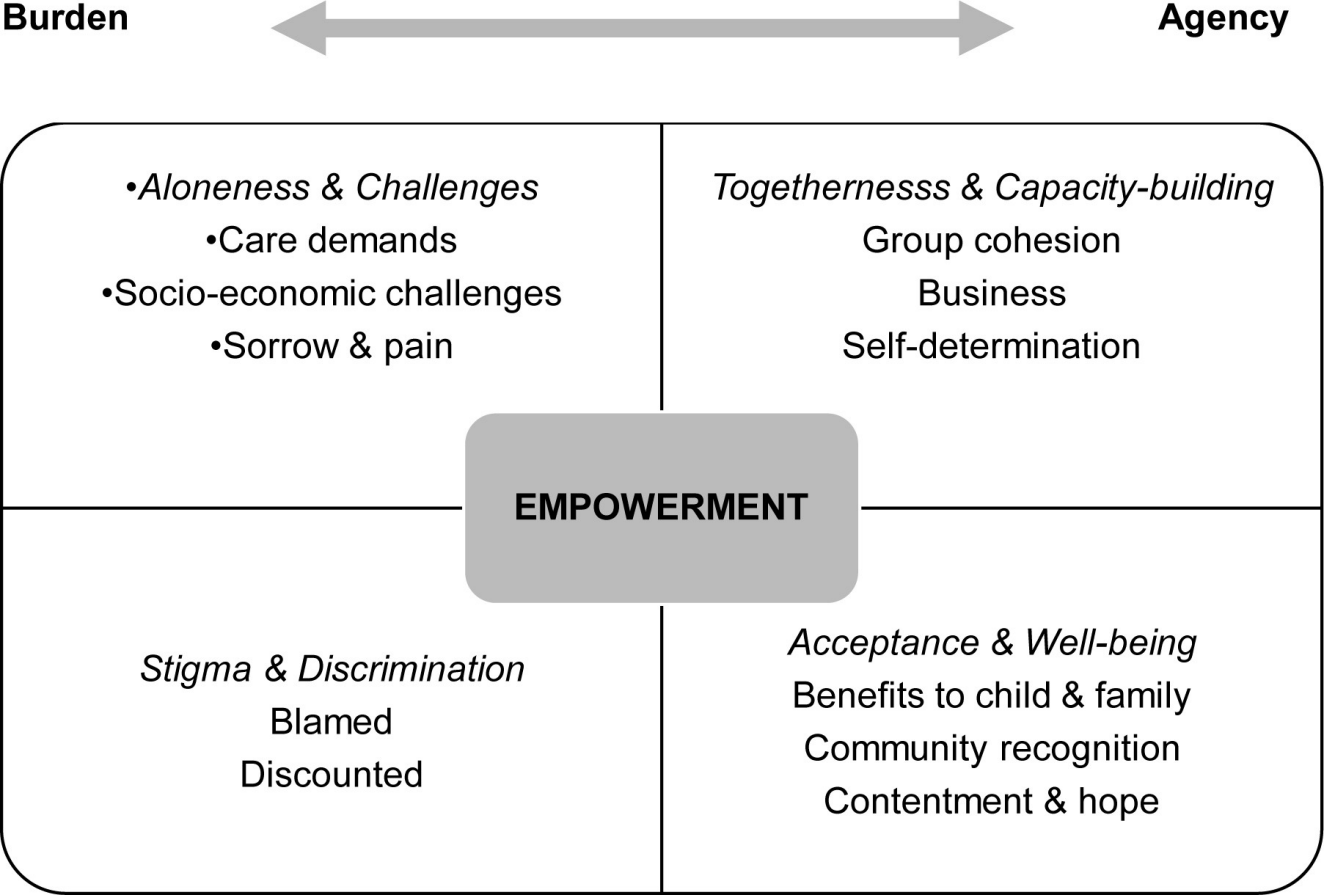
Schematic diagram illustrating the framework of thematic constructs.

As shown in Fig 1, **Burden** and **Agency** emerged as opposing constructs of a continuum that captured the possibility of movement between them. **Burden** represented the difficulties faced by the caregivers in bringing up a child with disabilities. Mainly present in the pre-intervention data, there were 138 references made, and only 4 references post-intervention. There were two organising themes: *Aloneness & Challenges*, which consisted of ‘care demands’, ‘socio-economic challenges’ and ‘sorrow & pain’; and *Stigma & Discrimination*, which consisted of ‘blamed’ and ‘discounted’. **Agency** captured the developing control exercised by group members and improvements to their quality of life. It emerged emphatically in the post-intervention data with 228 references, compared to 21 references at the pre-intervention stage. It was defined as *Togetherness & Capacity-building*, which included ‘group cohesion’, ‘business’ and ‘self-determination’; and *Acceptance & Well-being*, which included ‘benefits to child & family’, ‘community recognition’ and ‘contentment & hope’.

Empowerment is represented in the diagram (Fig 1) at the centre of the SHG activities. Whilst the majority of **Burden** Vs **Agency** themes were aligned to pre- and post-intervention time points respectively, there were examples of **Agency** at the pre-intervention stage also (n = 9 references). The data revealed multiple interconnections across the four quadrants of organising and basic themes.

The findings are presented in a series of four tables displaying organising (italicised text) and basic themes (filled bullet points) under the **Burden**—**Agency** continuum. Sub-ordinate themes are indicated as appropriate by unfilled bullet points. Supporting quotes are referenced by participant and SHG in square brackets, with pre- and post-intervention status indicated by a -1 or -2 respectively.

#### Aloneness & Challenges Vs Togetherness & Capacity-building

As shown in Table 4, the child’s problems and the associated care demands were talked about mainly at the pre-intervention stage, conveying the caregiver’s sense of **Burden.** Reference was made to the child’s inabilities, such as problems with mobility, vision, hearing and communication. They described the need to perform everyday tasks for the child that included washing, toileting, dressing and feeding. Concurrently, there was the absence of ideas and actions to improve the existing situation. The caregivers conveyed the sense of being alone, with care for their child falling squarely on their shoulders. Some even talked of their husbands ignoring the child. Post-intervention, renewed energies and purpose to the caregivers working together and engaging in mutual ‘problem-solving’ emerged. Capacities for ‘self-determination’ evidenced a growing sense of **Agency** amongst the caregivers who were driven to take control and act.

**Table 4:** Challenges & Aloneness Vs Togetherness & Capacity-building.

Burden	Agency
*Challenges & Aloneness*	*Togetherness & Capacity-building*
• Care demands ‘Even when he wants to help himself, I have to carry him or if I’m far from him he can just soil himself. Then I have to wash him.’ [CG082-1] ○ Child’s problems ‘She does not know how to sit properly, she does not hold her dress well leaving her sitting half-naked.’ [CG049-1]	• Self-determination ○ Problem-solving ‘… we sat together and found ways and means of how we could help each other . . . If we don’t (visit) we use mobile phones… my child has this and this. We call each other and visit the child. We discuss what we could do.’ [CG150-2]
• Socio-economic challenges ○ Money ‘I can’t leave to go and work because of how she is.’ [CG051-1] ○ Sustenance ‘… availability of food, sometimes I get, sometimes I miss to get the food. I have to struggle to get. There are also clothes.’ [CG229-1]	• Business ○ Income generation ‘…after making makuti, we sold them and then we divided the money at Christmas time. We contributed again here. We were to share that money, but we went and increased our stock; maize flour, wheat flour.’ [CG161-2] ○ Loans & savings ‘…you can borrow money from the group…’ [CG237-2] ‘Whatever we get, we send it there (to the bank). So we have the feeling that there (the bank) there will be more prospects.’ [CG112-2] • Self-determination ○ Decision-making ‘When we sit we plan our business, how is it progressing; if certain items are finished, we plan and then get more items for sale.’ [CG112-2]
• Socio-economic challenges ○ Support ‘I have to carry the child and do everything as if people are not seeing what I’m going through. Even when I sit and chat with them, I always feel lonely as I know duties are waiting for me to care for the child.’ [CG100-1] • Emotional impacts ‘I felt my heart was burning. I was not feeling good.’ [CG023-1]	• Group cohesion ‘It is the unity of the group. Every Tuesday we meet here to see how we could make better our lives.’ [CG080-2] • Self-determination ○ Problem-solving ‘It is not sitting there looking at your child with a disability without doing anything.’ [CG080-2]

Poor financial resources and inadequate clothing and food, time pressures and lack of local support exemplified some of the ‘socio-economic challenges’ described by the caregivers at the pre-intervention point. This was associated with feelings of ‘sorrow & pain’, that denoted their suffering. Post-intervention, talk of group ‘business’ activities and the associated financial benefits was in marked contrast. The previous lack of available ‘support’ and feelings of being alone with the challenges of caregiving transformed into togetherness, group cohesion, and pro-active problem-solving.

#### Stigma & Discrimination Vs Acceptance & Well-being

As shown in Table 5, stigma was portrayed in the negative images described by the caregivers, with discrimination as a by-product. The caregivers’ narratives revealed aversive responses by some people in the community, which were directed at the child and the caregiver. The caregivers spoke of how they were ‘blamed’ for giving birth to a child with disabilities. Examples included a suspected breach of social conventions, e.g. ‘inappropriate relations’ implicating incest or extra-marital conduct, or ‘traditional beliefs’ that told of punishment wrought through witchcraft or an angered God. Sometimes the caregiver was held accountable for other ‘undesirable events’, such as drought and the disappearance of food supplies. ‘Discounted’ covered the mistreatment or abuse of the child with disabilities, with actions ranging from verbal condemnation to actual physical harm. There were also direct acts by other people that prevented or stopped the child with disabilities taking part or being included in the community, i.e. exclusion. Some were denied access to shared activities or places, which resulted in the child being forced to eat and play alone.

**Table 5:** Stigma & Discrimination Vs Acceptance & Well-being.

Burden	Agency
*Stigma & Discrimination*	*Acceptance & Well-being*
• Blamed ○ Undesirable events ‘I just saw people gathering at my house…village leader said these people are here because of this child of yours. They are claiming that such children prevent rain from coming.’ [CG05-1] ○ Traditional beliefs ‘when you get a child of this nature, people think it is a curse, others think it is witchcraft, so you are rejected.’ [CG100-1] ‘They indicated that it was from us, that God was punishing us.’ [CG100-1] ○ Inappropriate relations ‘It is when someone comes and asks to hold your child, then begins to say that you have started bringing other tribes in the family…people with small heads . . .’ [CG229-1]	• Community recognition ‘The community used to say, ‘how is that child?’ But now they regard her as they do to normal children.’ [CG049-2] ‘The community is impressed. In fact some members are expressing desire to join.’ [CG235-2] ‘Even the neighbourhood, sometimes they show some kindness. But not always.’ [CG251-1] Inclusion ‘My child used to be left alone, could not mix with other children. This project has made my child in school mix with other children.’ [CG119-2]
• Discounted ‘…they say they will not treat a child who is already dying…I will not waste my money.’ [CG081-1] ○ Abuse ‘he can beat the child’ [CG252-1] ‘… they make fun of my child. I feel very bad.’ [CG122-1]	• Contentment & hope ‘I see how things are moving, I think they will produce benefits which will benefit my child.’ [CG112-2] ‘Despite the challenges I feel happy because I know that it is God who gave me this child.’ [CG229-1]

Post-intervention, there was positive notice of the SHGs by the local community, to the extent where caregivers reported approaches from people wishing to join their enterprise. This showed a positive form of ‘community recognition’, which extended to ‘inclusion’ as the caregivers told of their children with disabilities mixing with typically developing peers. This was the antithesis of the discrimination identified at the pre-intervention stage that was exemplified in the abuse meted out to the children. Whilst these negative behaviours may have persisted post-intervention, they were not mentioned. Instead, the caregivers spoke of their contentment, hopes and aspirations for their child. Positive feelings referenced pre-intervention, were typically connected to religious beliefs, which brought its own kind of acceptance as a form of coping strategy.

#### Aloneness & Challenges Vs Acceptance & Well-being

As shown in Table 6, the ‘care demands’ described initially revealed a dependent relationship between caregiver and child, with a focus on the problems that presented. Post-intervention this was connected with ‘benefits to child & family’ demonstrating a shift towards meeting the child’s needs in relation to ‘education’ and livelihood’. The caregivers described going to market to sell crops and rearing livestock. They talked of improved food supplies and clothing for the child. Some even spoke of older children contributing to the local economy through livestock ownership. This implied a sort of enabling relationship, as opposed to a caring for/being cared for relationship. Although benefits were also recognised pre-intervention, they were in the form of donations of food and clothing from religious organisations, e.g. the local mosque, and in relation to specific skills acquisition by the child, e.g. walking.

**Table 6:** Challenges & Aloneness Vs Acceptance & Well-being.

Burden	Agency
*Challenges & Aloneness*	*Acceptance & Well-being*
• Care demands ‘If it is eating, I have to feed her, if it is toileting, I have to help her. I have to carry her. I have to do everything for her.’ [CG100-1] ○ Child’s problems ‘…can’t talk and can’t hear…when he sits with others, he has a tendency of hitting others; he hits other children.’ [CG122-1]	• Benefits to child & family ○ Education ‘I then see what my child needs…school uniform, shoes and books.’ [CG049-2] ○ Livelihood ‘Even the child with disability also benefits…when I get groundnut for him, he ties them into packets and he sells.’ [CG072-2] ○ Child’s abilities ‘… my child can be sent to fetch items like spoon. That makes me happy. [CG082-1] ‘After going for therapy, I can see that she can now hold a cup, eat by herself.’ [CG081-1]
• Socio-economic challenges ○ Food, clothing & shelter ‘The really challenge is food, a place to sleep, I don’t have.’ (CG073-1] ○ Finance ‘…even if I want to take her for exercises, it becomes a big problem because I do not have even the fare to the hospital.’ [CG051-1]	○ Sustenance ‘Now he can get milk to drink. I can get other eatable things for him. He eats to increase his strength.’ [CG093-2] ‘I prepare good breakfast for her.…. I buy soap for her. Even clothes I do buy for her.’ [CG074-2] ○ Health ‘Because you have food, the money you get can assist in taking the child to hospital to get treatment.’ [CG173-2]
• Socio-economic challenges ○ Support ‘Like my husband does not like taking care of the child. If the child is sick does not seem to care.’ [CG252-1] • Sorrow & pain ‘I felt my heart was burning. I was not feeling good.’ [CG023-1] ‘I feel pain.’ [CG049-1]	• Contentment & hope ‘I feel a bit good in my heart. It is not as before.’ [CG235-2] ‘This child has given me hope. Even I have observed changes in the father towards the child.’ [CG119-2] ‘Every Tuesday we meet here to see how we could make better our lives.’ [CG080] ‘That he goes to school and maybe his future life may be good.’ [CG252-1]

The ‘socio-economic challenges’ of pre-intervention spoke of poor or inadequate resources in contrast to the improved supply of food and clothing, and money for transport to access health facilities. More than one caregiver identified a lack of ‘support’ from within the family, which was associated with their own emotional pain. This connected with growth in their expressed ‘contentment & hope’ at post-intervention. The caregivers started to express their aspirations in relation to not only the future of their child with disabilities, such as schooling, but also the SHG’s activities, demonstrating renewed hope and also ambition.

#### Stigma & Discrimination Vs Togetherness & Capacity-building

As shown in Table 7, being ‘discounted’ revealed community responses that treated the child with disabilities differently and in a discounted way, leading to their ‘exclusion’. Such acts were associated with the psychological impacts on the caregiver, where expressed sadness was linked to the aversive responses of others: “My child being looked down upon makes me very sad.” [CG254-1]. ‘Group cohesion’ and ‘self-determination’ afforded by the SHG process formed a counter strategy that brought caregivers and their children together with developing capacities and peer support to answer such negative acts.

**Table 7:** Stigma & Discrimination Vs Togetherness & Capacity-building.

Burden	Agency
*Stigma & Discrimination*	*Togetherness & Capacity-building*
• Discounted ○ Exclusion ‘People at home or even my co-wives when they see him they close their doors, or even chase him away saying, ‘Go to your mother.’ He comes back or sits at the door because he does not understand himself.’ [CG081-1]	• Group cohesion ‘…. we thought if we sit together we could benefit with our children.’ [CG112-2] ‘What has kept us together is communication. What are we going to do, what are the best ways to follow, then we do together. We understand each other, otherwise you would not have seen us together.’ [CG121-2] • Self-determination ○ Problem-solving ‘The secret is that we identify the problems our children have and try to solve them ourselves.’ [CG155-2]

### Triangulation and underlying mechanisms

The distinction between **Burden** Vs **Agency** captured in the expressed views of the caregivers pre- and post-intervention corresponded to the growth in perceived social support networks. Heightened awareness of *Stigma & Discrimination* and *Aloneness & Challenges* at the pre-intervention stage contrasted with *Acceptance & Well-Being* and *Togetherness & Capacity-Building* post-intervention. Although the child’s severity of disability was unlikely to have resolved, the post-intervention perception of it being less severe is likely associated with the more positive views expressed by the caregivers. Similarly, extrinsic factors affecting the caregiver and child were viewed as significantly less of a problem, which may be associated with different emphases on **Burden** and **Agency** at the two time points.

Two mechanisms appeared to be critical to the group processes: handling goods and money; and social ties and support. Handling goods and money formed a regular activity in all the groups and was largely evidenced in ‘business’, as shown in Fig 1. (post-intervention: 60 references). It captured activities in relation to the exchange of goods and money, group subscriptions and loans, counting up and recording transactions, income generation and savings. There were strong links to ‘benefits to child and family’ and ‘contentment and hope’. The mechanism of social ties and support was substantiated by ‘group cohesion’ (post-intervention: 29 references; pre-intervention: 1 reference), where the caregivers talked of working together on their enterprises; and ‘self-determination’, which captured shared decision-making about business-related matters and mutual problem-solving in difficulties variously affecting the caregivers and their children (27 references post-intervention only). There were connections to ‘community recognition’, where ties to the wider community were starting to develop, and ‘contentment and hope’, where the caregivers reflected not only on their own positive state of mind, but on improvements shared with other members of the group.

## Discussion

The aim of the current study was to determine the outcomes of caregiver participation in SHGs and the underlying mechanisms associated with changes and differences pre- and post-intervention. The perceived **Burden** of caregiving emerged in the pre-intervention interviews where the caregiver experience was characterised by *Aloneness & Challenges* and *Stigma & Discrimination*. A sense of **Agency** was expressed emphatically post-intervention, which was defined by *Togetherness & Capacity-building* and *Acceptance & Well-being*. This was consistent with significant gains in caregiver assessment of their social support, and significant decreases in the perceived severity of their child’s disability and in the impact of extrinsic factors on the caregiver and child.

The challenges of bringing up a child with disabilities were all pervasive, affecting caregiver management of domestic arrangements and their psychological wellbeing. The low mood and sense of helplessness expressed by the caregivers was associated with a lack of support, both within the family and externally in the community as reported previously [5,6, 10, 17, 24). Indeed, the common experience was one of psychological stress and daily challenges, which concurs with the work of Masulani-Mwale [26]. The burden of caregiving appeared to be related to a lack of agency or control over the events that challenged on a daily basis. Zimmerman and colleagues [31–34, 58] might explain this as the antithesis of empowerment. Having a child with a disability was considered generally incongruent with acceptability in the prevailing culture, and indeed triggered negative responses from others in the family and in the community as reported previously [4,5, 17–19, 21–26]. Thus both caregiver and child were effectively marginalised in their own community, with a lack of knowledge, limited support and no opportunity for joining their efforts with others–critical components of the empowerment construct described by Perkins & Zimmerman [58].

The set-up of SHGs brought the caregivers together in a movement that was counter to their isolation. The process of identifying themselves in relation to a shared characteristic, that of being a caregiver to a child with disabilities, supported group formation, which was similarly observed in Ghana [5]. The facilitated intervention invited the sharing of personal narratives in a safe environment that was characterised by acceptance and shared experiences in common. A component of the empowerment process, speaking up, was indirectly linked to a developing self-concept and acting for change, consistent with Moran et al [30].

The post-intervention interviews demonstrated compassion and responsiveness amongst the SHG membership as relationships formed. Participating in the SHG not only offered new social connections, but also created a kind of buffer to the harmful effects of parenting stress and isolation as described by Thoits [15]. Not surprisingly, the caregivers reported a greater sense of their own well-being, with more than one caregiver attributing positive changes in their husband to their own participation in the SHG. Whilst a causal relationship between their participation and attitude changes in the family cannot be proven, it is possible that growing confidence in the caregivers was recognised by marital partners and other family members, as reported previously [5, 41,42]. The early address of income generation activities helped to grow capacities amongst the SHG membership. Through their active participation of SHG meetings and the chosen livelihood activities, the caregivers learned to initiate actions and contribute to the group’s work. This corresponds to the *intrapersonal* dimension of empowerment whereby the caregivers became aware of their ability to influence change, combining their efforts in pursuit of income generation [34]. Establishing a common goal for the caregivers would not only have supported the empowerment process but produced some direct benefits for the caregiver’s family. This is consistent with report from Ghana and India [5, 38, 43,44]; as suggested previously, where benefits included greater food security and clothing. Such economic improvements were associated with SHG participation, which was formed on the basis of having a child with disability. Thus caring for a child with a disability was also linked to the changing fortunes of the family.

Closely connected to this was the caregiver’s changing perception of their child’s disability and their personal situation. The caregivers perceived their child’s disability as less severe, which is consistent with Bunning et al [59], even though no direct intervention was applied to the children. Furthermore, this extended to the caregiver’s revised view that there was sufficient time and support to carry out their domestic duties. These altered perceptions were possibly artefacts of the linkage between growth in social ties and support, and the mental health and well-being of the caregivers consistent with Thoits [15]. Rather than participate in other SHGs focused solely on economic empowerment using shares, savings and micro-finance, the groups were initially formed on the basis of a shared experience. Growth in social networks was a bi-product of being part of a group. Many of the members emphasised the importance of communication amongst their peers. In addition, the caregivers were encouraged to talk with each other about their own personal situations in a safe environment. This represented a counterpoint to the more usual protective responses developed to the negative attitudes espoused in the local community. The caregivers participated in active debate about issues related to their caregiving role, which established common ground and shared experiences amongst the SHG membership. This adoption of new norms and behaviours is consistent with empowerment theory and connected to the *interactional* dimension of empowerment as determined by Zimmerman and colleagues [31–34, 58]. Beyond within-group interactions, the caregivers recounted interactions in the neighbourhood and engagement with services in the health and educational sectors. Thus their *interactional* space was observed to extend.

Underlying mechanisms determining the outcomes appeared to relate to handling goods and money, and social ties and support. Similar to the economic and livelihood groups reported from South Asia [14, 40,41], early engagement in income generation activities, involved all members in handling goods and money, whether it was small bags of maize flour and rice, or Kenyan shillings as their weekly contributions to the group. Every meeting started with collecting and recording the week’s subscriptions, and whilst the elected roles of chair, secretary and treasurer led the transactions among the membership, the process involved all the members. Before the establishment of group bank accounts, some of the groups used a special bag in which to keep the group’s savings, such as a silver handbag. This demonstrated the value placed on this activity. The act of participating in financial/goods transactions as part of an in-group managed process provided a tangible purpose to the SHG meetings and helped to grow the capacities of the membership. Although the group officer roles were responsible for receiving, counting, recording and distributing member contributions, all the members were involved in group transactions. Member actions accorded with an agreed set of rules for Merry-go-round and group start-up projects. Despite their limited educational backgrounds, the caregivers developed skills in numeracy, recording and financial/goods management. Thus the caregivers acted together to bring about change, which included the handling of goods and money in group meetings, but also working together in chosen livelihood activities. This relates to the *behavioural* dimension of empowerment [34]. Furthermore, recognition of their own exercise of skills in relation to a valued activity offered the caregivers a revised view of themselves [31]–one of taking control, which resonates findings reported by Moran and colleagues [35].

Caregiver isolation was challenged by meeting others in similar situations providing opportunities to develop new social connections. In a sense, each caregiver saw something of their own life’s experiences in the stories shared by others, which enabled a social tie. This process of recognition is what the German philosopher Axel Honneth considered a pre-requisite to empathy [57]. Deliberate encouragement was given to the caregivers to speak out in the groups, sharing their personal situations and reviewing opportunities for peer support. Thus by recognising themselves in others, the caregivers were helped to rationalise their own experiences, to express their sense of aloneness and to challenge the existing situation. Social support refers to the functions served by the members of the SHG in relation to each other. Encouraged by the facilitated intervention, the members talked of assisting each other both practically and emotionally. The caregivers learned to initiate change through their active participation of the SHGs, which corresponds to the *intrapersonal* dimension of empowerment [34]. Their collective action reflected a basic component of the empowerment construct as described by Perkins & Zimmerman [58], serving to move them towards a greater sense of ‘agency’. Inter-connected, the structural ties of the group enabled support in terms of mutual problem-solving, shared decision-making and deliberate action.

### Limitations

The rural distribution of the groups and participants, their availability at the time of the pre-arranged research visit, project time and financial resources meant that convenience was a major factor in the samples for each measure. Whilst saturation checks on the analysis of interview data were carried out, limited project resources meant that follow-up interviews could not be planned A lack of tools for measuring impacts that had been standardised on the population meant that quantitative instruments had to be devised for use. Whilst the CDP had been developed in Uganda and used in previous studies in Kenya [59], the MSPSS [53], originally developed in Europe, had to be adapted, translated, back-translated and piloted.

## Conclusion

This research provides a proof of concept for the development of empowering SHGs for caregivers of children with disabilities: they can be facilitated and have been shown to have positive outcomes. As a counter response to the various stresses and challenges experienced by caregivers, including poverty, lack of psycho-social support, and stigma originating from the community, the SHGs provided a route to empowerment. Not simply about identifying the rights of an individual, the SHGs provided a space where the burden of caregiving could be challenged. Through development of a social structure and resources, and the establishment of a degree of social connectedness, the caregivers were enabled to make decisions and to take actions, to demonstrate abilities and to engage in capacity-building. The mechanisms of handling goods and money and social ties and support appeared to be important determinants of the outcomes. Through participation in such activities, the caregivers gained a better understanding of factors affecting their lives, extended their knowledge of available resources to achieve goals, and developed skills for decision-making and problem-solving. Ultimately, the caregivers started to think differently about their own capacities to influence change. Thus empowerment was associated with caregiver participation in SHGs .

Documented processes and reported outcomes associated with the SHG development contribute to the evidence on community-based inclusive development. The data have informed a model of how to set up SHGs that is replicable in other low-income settings. The value of SHGs as empowerment-focused structures is demonstrated in their influence on the CBR domains of ‘health’, ‘education’, ‘livelihood’ and ‘social’. Caregiver-driven processes and actions support burgeoning agency without the need for extra specialist resources such as the engagement of professional practitioners. Through participation with others, the caregivers are helped to take control of their lives and the lives of their children in ways that are meaningful and sustainable.

## Supporting information

S1 Appendix Topic guide with questioning route. (DOCX)
