# Supplementary material for: Empowering self-help groups for caregivers of children with disabilities in Kilifi, Kenya: Impacts and their underlying mechanisms
Source: PLoS One. 2020 Mar 9;15(3):e0229851. doi: 10.1371/journal.pone.0229851 (PMC7062261; doi:10.1371/journal.pone.0229851)
Supplement: S1 Appendix — (DOCX) [file pone.0229851.s001.docx]

**Appendix i Topic Guide with questioning route**

Guiding Questions before facilitated intervention

1. What is the problem with your child?
2. What challenges do you face?
3. What assistance do you get from relatives/friends/neighbours
4. How does the community assist you?
5. What good things have happened to your child recently?
6. What about things you did not like?

Guiding Questions after facilitated intervention

1. Last time I asked you about how you felt about your child with disability, how are things now?
2. What has helped?
3. What has made the group stay together?
4. How has the community helped?
5. How has your child with a disability benefited from this project?
